# Variola

**Published:** 1872-05

**Authors:** A. S. Payne

**Affiliations:** Virginia


THE GEORGIA

### MEDICAL COMPANION:

A MONTHLY ADVISER.

VOL. 2. ATLANTA, GA., MAY, 1872. No. 5

### PART I.

### Original Communications and Special Selections.

### VARIOLA.

BY A. S. PAYNE, M. D., OF VIRGINIA.

I have thought it might not prove uninteresting to your numerous readers for me to give, through the columns of THE GEORGIA MEDICAL COMPANION, a short history of Variola, or Small-pox, as it occurred in Clark and Fauquier counties during the late war. In saying that I recognized this disease by the pulse, before I ever dreamed of its existence amongst us, and before any eruption whatever had made its appearance, I do not wish to be understood as claiming for myself any particular astuteness, but rather to show that this disease has, in my opinion, a pulse of its own—i. e. "sui generis."

I was called to see Albert Elsea, on the 27th day of October, 1863, residing on the eastern bank of the Shenandoah River in Clark county. The day was cold and damp, and as I sat in the darkened room by the fire, asking him questions preparatory to a minute physical examination, I became satisfied in my own mind I had a severe case of pneumonia, or rather apoplexy of the lungs, to deal with. I learned from him that, some six days before, he had been over to Millwood, located on the west side of the Shenandoah, and returning had been exposed to a very heavy rain. His face was flushed, respiration hurried and laborious, with excruiating pain in the small of his back. After becoming comfortably warm, and getting through with my questioning, I advanced to his bed-side and placed my hand upon his pulse. Had I placed my hand upon a snake I could not have been more startled. I then, too, for the first time, saw lying in the same bed, behind his father, a little boy of some seven or eight years of age, with the same breathing and the same flushed countenance. I felt the boy's pulse, it was the same peculiar one as that of the father's. I paused for a moment and put the question to myself, where have I felt this pulse before? Although I have practised my profession for seventeen years in the State of Virginia, I certainly have never crossed such a pulse as this before in Virginia. It is true I have felt it before; now, where was it? Instinctively it flashed in my mind, "the last time you felt this pulse was in the Spring of 1846, at the 'Five Points,' New York city, and you have often felt it before that time in the Small-pox hospital in New York." "Al., have you ever been vaccinated?" "Yes, but it has been over twenty-five years ago." "Have any other members of your family been vaccinated?" "No." "You had better be re-vaccinated, and the rest of your family vaccinated as soon as possible." I prescribed for both patients, and had just mounted my horse (wishing to avoid the questioning of the good wife) to ride off when Mrs. Elsea hailed me: "Doctor, what ails Al.?" "I have tried to get off without seeing you, to avoid answering that *very* question; but if you must know, I am truly, truly sorry to tell you he has the Small-pox. I am going to hunt some vaccine matter and will be back to-morrow to vaccinate you all, although I fear it is too late to do much good. Good evening."

At Mt. Carmel, midway between Mr. Elsea's and my own residence, there was in active progress a Methodist protracted meeting. I rode up to the door, called the ministers and elders of the church out and told them the Small-pox had broken out some four miles below the church, I believed it would prove to be of a malignant type, and I wished they would "break up" the meeting. A good many of my most intimate acquaintances among the congregation were disposed to make merry at my expense, saying it was nothing but the "chicken pox." "Well, dismiss the congregation and laugh at me afterwards should it prove to be only the chicken pox." Though they laughed, yet I noticed men, women and children did their own saddling. Miss W—, a young lady, eighteen years of age, living on the west side of the Shenandoah, and immediately on the opposite bank from Mr. Elsea's, was present at that meeting having left her mother and father at home, both sick with what was supposed to be "chicken pox." This young lady and her father both died with Small-pox. And, on her return home, she came by Mr. Elsea's, where I saw her, and felt her pulse, just two days from the time this meeting was disbanded. On my arrival at home, I went straight for my office and had a suit of clothes thrown in, before I dared to present myself to Mrs. P—. After this precautionary measure was adopted, even then, it was amusing to see how Mrs. P. and the young bairns would shy me. The next day I rode over in the neighborhood of Berryville, to procure some vaccine matter. Dr. Randolph, of Clark, gave me some, (it was old and proved worthless), and requested me to stop and see a child of Mrs. Lloyd, just below Millwood, that he said had some eruptive disease. I had passed the house, but Mrs. Lloyd recognized me, as she had once lived on my side of the river, and I had been the family physician. As soon as I saw the child, I pronounced it a mild case of confluent small-pox. She then said, "if this child has the Small-pox, my other child, that died a week or two ago, had the same."

TO BE CONTINUED.

